# Student teachers’ perceptions of flipped classroom in EFL teacher education

**DOI:** 10.1007/s10639-023-11839-w

**Published:** 2023-05-01

**Authors:** Han Han, Fredrik Mørk Røkenes, Rune Johan Krumsvik

**Affiliations:** 1 Department of Teacher Education, Norwegian University of Science and Technology, Gunnerus Gate 1, 7012 Trondheim, Norway; 2 Department of Education, University of Bergen, Bergen, Norway

**Keywords:** Flipped classroom, Student teachers, Digital competence, Post-Covid-19

## Abstract

**T**his paper aims to provide evidence on student teachers’ perceptions of Flipped Classroom (FC) to help teacher educators (TEs) to make informed decisions about implementing FC and support student teachers to reflect on the value of FC in their teaching practice. FC, a pedagogical model requiring digital competence of students and teachers, has been a popular teaching approach for nearly two decades in K-12 and higher education. After the outbreak of Covid-19, more teachers have started to implement FC. In post-Covid-19, with the possibility of reusing video lectures made during the pandemic and the familiarity of digital skills to create digital lectures, a question for teachers is whether to continue with this approach. This paper follows an explanatory sequential mixed methods research approach. Insights from student teachers (STs) in the field of English as a foreign language (EFL) in Norway are the primary data, and surveys and focus group interviews are the main instruments to collect the data. FC’s advantages and challenges perceived by STs are reported, and the possibility of STs becoming future flippers is explored. Findings from this paper indicate that STs would like to have more courses flipped in their studies, yet STs seem hesitant about flipping their courses in their teaching practice. STs also provide some practical suggestions on implementing the FC approach.

## Introduction

Flipped Classroom (FC) has been a popular teaching approach for nearly two decades in K-12 and higher education (Han, & Røkenes, 2020; Van Alten et al., 2019). Different from a *chalk and talk* way of teaching in which teachers write on a blackboard (with chalk) and lecture whole classes, with the FC approach, “students study instructional material before class (e.g., by watching online lectures) and apply the learning material during class” (Van Alten et al., 2019, p. 1). The theoretical underpinning of FC is centered on “student-centered learning theories based on the works of Piaget 1967 and Vygotsky” (Bishop & Verleger, 2013, p. 5). Research has revealed that with the support of information and communication technology (ICT) (Zheng et al., 2020), FC can improve students’ learning achievement, active learning, high-order thinking, motivation, engagement, and ease students’ anxiety (e.g., Akçayır & Akçayır, 2018; Bergmann & Sams, 2012; Dove & Dove, 2017; Meyliana et al., 2021). However, some teachers have been reluctant to adopt FC for various reasons such as possibly increasing preparation time or lack of professional digital competence (Røkenes et al., 2022; Meyliana et al., 2021; Polly et al., 2018).

Nevertheless, after the mode of teaching was forced to change from face-to-face to fully online due to Covid-19, researchers indicate that both teachers and students have adapted to mere digital teaching and learning environments (Khlaif et al., 2021), and more teachers have started to implement FC (Collado-Valero et al., 2021). In post-Covid-19, with the possibility of reusing video lectures made during the pandemic and the familiarity of digital technologies for creating digital lectures, a question for teachers is whether to continue with the FC approach and take it as the new normal in teaching. Students’ thoughts on FC can be helpful for teachers to make informed decisions about whether and how to implement FC. There is ample research on students’ perceptions of FC (e.g., Adnan, 2017; Conner et al., 2014; Fraga & Harmon, 2014; Van Wyk, 2018), yet these studies were mainly conducted before the outbreak of Covid-19. Currently, limited published studies have explored students’ perceptions of FC after the pandemic. In response to this need, this study aims to explore student teachers’ perceptions of FC after the outbreak of Covid-19 by providing evidence from student teachers (STs) studying at a teacher education program in Norway. STs are students learning to be teachers in the future. On the one hand, as students, STs’ perceptions of FC can provide useful information for teacher educators (TEs) to consider whether and how to implement FC in teaching (Cabi, 2018; Fraga & Harmon, 2014). On the other hand, as potential teachers-to-be, STs’ insights into FC can indicate whether FC will be applied in primary and secondary education (Graziano, 2017). This study intends to discuss two research questions:

1. What are student teachers’ perceptions of Flipped Classroom regarding advantages and disadvantages?
2. To what extent do student teachers prefer Flipped Classroom, and what are their suggestions for its future implementation?

## Background

This section first describes the definition of FC and the connection between FC and digital competence. Second, social constructive theory as the theoretical framework is discussed regarding the relation to FC. Finally, previous research on STs’ perceptions of FC is presented and linked to the purpose of this study.

### Flipped classroom and digital competence

Several researchers and practitioners have proposed definitions to capture the essence of FC. Lage et al. (2000), without proposing the term, provided a simple definition of the inverted (or flipped) classroom: “Inverting the classroom means that events that have traditionally taken place inside the classroom now take place outside the classroom and vice versa” (Lage et al., 2000, p. 32 Saldaña). Bishop and Verleger (2013), however, argued that the explanation of Lage et al. (2000) did not “adequately represent the practice” (p. 5) of FC. Therefore, they highlighted two aspects of FC’s activities: “interactive group learning activities inside the classroom” (p. 5) and “direct computer-based individual instruction outside the classroom” (p. 5).

FC involves using ICT for teaching and learning, and thus, implementing and taking advantage of FC requires digital competence in teachers and students. The European Commission (2019, p. 10) notes that digital competence “involves the confident, critical and responsible use of, and engagement with, digital technologies for learning, at work, and for participation in society”. When designing lessons using an FC approach, teachers often need to prepare video lectures as “direct computer-based individual instruction” (Bishop & Verleger, 2013, p.5), involving “talking head” lectures, voice-over PowerPoint presentations, or interactive video lectures with embedded quiz sections. In addition, teachers must design learning activities (with or without ICT) for students to work on the subject discipline during in-class time. Consequently, following an FC approach requires teachers to possess pedagogical or professional digital competence (PDC). The teacher’s PDC can be understood as “proficiency in using ICT in a professional context with good pedagogic-didactic judgment and his or her awareness of its implications for learning strategies and the digital Bildung of pupils and students” (Krumsvik, 2011, pp. 44–45). A deep understanding of digital technologies in teaching and learning beyond technical proficiency is an important part of PDC (Lund et al., 2014). While students seem to only need to know how to open and watch the video lectures that their teachers have produced, they also need digital competence involving “basic digital skills” (Røkenes & Krumsvik, 2016, p. 3), such as using learning management system, digital devices, and interactive learning tools. Students’ digital competence usually refers to “skills, knowledge, creativity, and attitudes required to use digital media for learning and comprehension in a knowledge society” (Røkenes & Krumsvik, 2016, p. 2; Erstad et al., 2021). In teacher education, when TEs are modeling good pedagogical practice with technology in FC, STs also develop their PDC in the dimension of “Didactical ICT-competence” (Røkenes & Krumsvik, 2016, p. 3). Meanwhile, STs start to see how they can implement FC in their teaching practice.

Although post-Covid-19, teachers and students are returning to campus and physical classrooms, promoting PDC and providing quality digital teaching still needs to be emphasized in educational research, also for preparing teachers and students for future scenarios (Olofsson et al., 2021). In teacher education, developing STs’ PDC also need to be continous effort to increase the quality and contribution of ICT training to their ICT self-efficacy (Guðmundsdóttir & Hatlevik, 2018). Reports from the Norwegian Agency for Quality Assurance in Education (Bakken, 2022; Wiggen, 2022) showed that few newly graduates from teacher education considered themselves digitally competent enough to master the digital forms of teaching in schools.

### Flipped classroom and social constructive theory

In an FC approach, teachers use classroom time to work as facilitators instead of lecturing, and use “interactive group learning activities” (Bishop & Verleger, 2013, p. 5) to provide a student-centered learning space to promote students’ learning. Student-centered or active learning theories look primarily to social constructive theory. According to social constructivism, learning occurs through social interaction and the help of others, including peers and teachers. In addition, when teachers follow a social constructivist teaching approach, they need to shift their role from “sage” to “guide”. With the FC approach, students learn through discussing or solving problems with their peers inside the classroom, with the knowledge they acquired from watching video lectures and working on other materials outside the classroom. Meanwhile, inside the classroom, students learn by asking questions, receiving guidance, collaborating in groups, and working on materials related to the subject disciplinary content.

### Purpose of study on student teachers’ perceptions of flipped classroom

With the FC approach, students’ roles have also change from passive receivers to active learners (Bergmann & Sams, 2012). Several researchers have started examining students’ thoughts on FC in a teacher education context (e.g., Conner et al., 2014; González-Gómez et al., 2016), and how STs perceive FC has drawn researchers’ attention in teacher education (Han & Røkenes, 2020). González-Gómez et al. (2016) reported that STs found FC useful for achieving learning objectives and improving engagement. In the study of Ng (2018), all the STs liked FC. In addition, FC was associated with “a general positive perception” (Jeong et al., 2018, p. 163) from STs not only in the face-to-face learning environment but also in the online environment (Van Wyk, 2018). Yet, there were STs who perceived FC negatively (Conner et al., 2014), and STs complained about more responsibility with the FC approach (Dove & Dove, 2017; Graziano, 2017). Besides responsibility, Fraga and Harmon (2014) found that STs mainly disliked FC due to two reasons: “issues of time management and confusion” (p. 22).

Whether STs, who have experienced FC in teacher education, would like to take more FC courses and implement the FC approach in their teaching practice is another interesting topic for researchers in teacher education (e.g., Dove & Dove, 2017; Jeong et al., 2018).

In light of previous research, STs seemed to favor having more FC courses in the future (Jeong et al., 2016, 2018). However, for future implementation of FC in their teaching, STs seemed to have different opinions. Many STs planned to flip their classrooms in the future (e.g., Graziano, 2017; Kurt, 2017). Yet, there were other varying thoughts as well. For instance, one ST commented, “I will not have the time during my first few years of teaching to accurately gather or make videos on my own” (Graziano, 2017, p. 124).

Students’ suggestions are helpful for teachers to improve teaching pedagogy, and researchers in teacher education should be interested in their input (e.g., Cabi, 2018). The STs in Conner et al.’s study (2014) gave several practical suggestions, including preparing questions for students to answer or offering “a set of partially completed notes” (p. 73) for students to “fill in the blanks” (p. 73) while watching online videos and increasing the interaction between students and teachers during the in-class time. In Adnan’s (2017) study, the STs suggested that since students might not be familiar with the FC approach, “students should be clearly informed to understand the flipped classroom model” (p. 220). Therefore, teachers should explain to students what FC is and what students are expected to prepare with the FC approach.

Given previous research, surveys, questionnaires, and focus group interviews were the most used instruments to explore STs’ perceptions. Employing surveys and focus group interviews in one study may provide a better understanding of STs’ thoughts due to the methods’ “potential complementary strengths” (Johnson & Christensen, 2017, p. 51). This study adopts both surveys and focus group interviews as the primary instruments to collect the data. Furthermore, the abovementioned previous studies were conducted before the outbreak of the Covid-19. Therefore, it is valuable to examine STs’ perceptions post-Covid-19 and explore whether they want to have more FC courses and implement the FC approach in their teaching career.

## Method

This study implements an explanatory sequential mixed methods design (Creswell & Creswell, 2018) and analyses both quantitative data and qualitative data (Johnson & Christensen, 2017) (see Table 1). In this section, the setting of FC context in this study is described first. Table 1 shows the participants in this study and instruments for collecting data are clarified.

**Table 1:** Research design, data-collection instruments, and participants

Explanatory Sequential Design	Data-collection Instruments	Number of Participants
Phase 1 (quantitative data)	Survey on Perceptions of FC	N_(Survey)_ = 34
	Exit Tickets from Each Session	N_(Exit Ticket)_ = 143
Phase 2 (qualitative data)	Focus Group Interviews	N_(Interview)_ = 19

### Setting of flipped classroom context

An obligatory course focusing on English linguistic knowledge was taught with the FC approach at a large teacher education program in a Norwegian university in autumn 2020. This course was scheduled with five physical teaching sessions over one academic semester and four hours for each session. However, due to the outbreak of Covid-19, the participating university took preventive measures where STs were divided into smaller groups (10–19 STs in each group) to socially distance in the classroom. The in-class time for each session was reduced from four to two hours. Due to the Norwegian Covid-19 situation, the first four sessions were conducted physically, while the last session was taught using a hybrid solution.

**Fig. 1:**
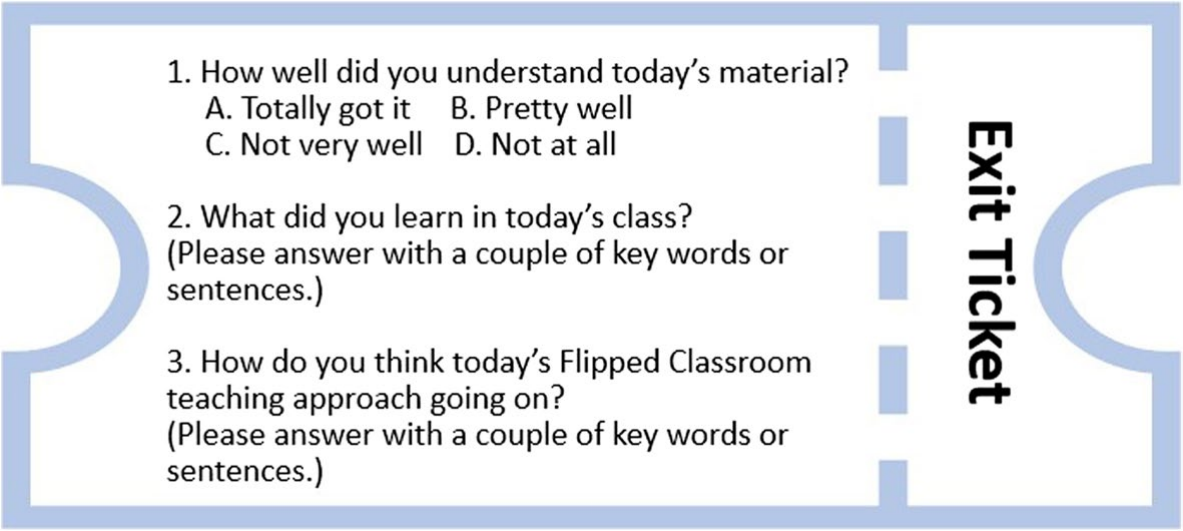
Exit ticket

The TE of this course pre-recorded six video lectures and posted corresponding ones to the learning management system about one week before each session. Besides viewing video lectures, STs needed to read from the reading list offered by the TE and work on obligatory written assignments as their out-of-class activities. As for the two-hour in-class activities, group discussions and pair or group activities were the main formats. To answer STs’ common questions or clear up general misunderstandings, the TE also had mini-lectures in the classroom. To gain rich information from the teaching sessions, the first author of this study acted as a non-participating observer in the classroom, taking field notes about in-class activities and collecting exit tickets (see Fig. 1) after each session. The field notes showed that the in-class time was mainly devoted to STs’ activities, because based on the classroom observation, over 50% of the in-class time was allocated to group discussion.

### Participants

The participants who took the required course in this study are second-year English as a Foreign Language (EFL) STs qualifying to teach grades 1–7 and 5–10.^1^ As can be observed in Table 1, 34 participants completed a survey in Phase 1, and 19 participants joined in focus group interviews in Phase 2. In addition, after each session, all STs taking the course were invited to fill in an exit ticket (see Fig. 1). 143 exit tickets in total were collected in Phase 1 for this study.

### Instruments for collecting data

To understand how STs perceive FC and what their suggestions are for implementing the approach, data were collected using a survey on STs’ perceptions of FC, focus group interviews with STs, and immediate feedback from their exit tickets.

#### Survey on perceptions of flipped classroom

To investigate STs’ perceptions of FC, a paper-based survey was developed by the authors, piloted with five EFL graduates, and then revised. This survey consisted of 19 questions in English using a five-point Likert scale and six open-ended questions (see Appendix 1). The participants completed the survey right after their last session of the course.

#### Focus group interviews

19 STs (13 females, six males) participated in focus group interviews (Merriam & Tisdell, 2016) after they completed the course but before taking the course exam. Each interview (see Appendix 2 for the interview guideline), between five to seven participants and the first author, was physically conducted in English and lasted for 45–60 min. In total, there were three focus group interviews.

#### Exit tickets from each session

To obtain immediate feedback from STs, all EFL STs taking the course were invited to voluntarily answer a three-question exit ticket after each session (see Fig. 1), and 143 exit tickets were collected in total through the digital quiz software Socrative.^2^ Three questions concerned how STs understood sessions’ materials, what they learned from sessions, and what they thought about the FC approach.

### Data analysis

Descriptive statistics were applied to analyze the survey responses to describe the most frequent answers and display the distribution of different replies. Word frequency on the collected exit tickets was counted.

Thematic analysis was used to analyze the interview data and the responses to the survey’s open-ended questions, aiming to explore STs’ shared perceptions of FC. The qualitative data were imported and analyzed using NVivo 12. Following Braun and Clarke’s step-by-step guide for thematic analysis (Braun and Clarke, 2006), the analytical process is recursive, with movements back and forth between the six steps. According to Saldaña (2016), a code is “a word or short phrase that symbolically assigns a summative, salient, essence-capturing, and/or evocative attribute for a portion of language-based or visual data” (p. 4). The approaches of coding and categorization, sub coding, and pattern coding in Saldaña (2016) were adopted during the analytical process to answer the research questions proposed.

## Results

### Student teachers’ perceptions of flipped classroom

STs’ perceptions of FC were explored by analyzing both the quantitative and qualitative data from the survey, focus group interviews, and exit tickets. The analytical process concentrated on the advantages and challenges of FC as perceived by the STs.

#### Advantages of FC perceived by student teachers

Among 34 survey participants, 91.18% of STs reported that FC used class time more efficiently. 85.29% of STs stated that they learned better and more effectively with the FC approach, and 79.41% of STs acknowledged that improving learning performance was one of FC’s advantages. Through open-ended survey questions, the STs explained that FC could take advantage of class time efficiently because by watching video lectures at home they might “get a taste of the material beforehand” and “come to class more prepared”. Meanwhile, the STs argued that TEs “spend less time explaining easy material” as to “free up time for deeper learning”, and therefore, the STs “can use class time on discussions and reflections”. Furthermore, they also stated that having their TE use more time walking around the classroom to “answer difficult questions” was helpful.

**Fig. 2:**
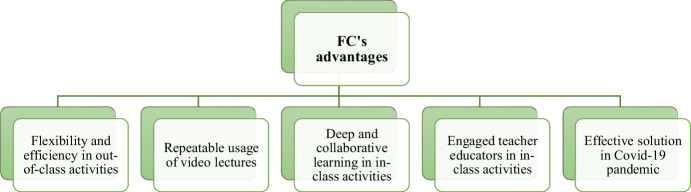
Advantages of flipped classroom

In the focus group interviews, the participating STs stated their perceptions of the advantages of FC, which were categorized into five themes (Fig. 2).

##### Flexibility and efficiency in out-of-class activities

The STs found that FC afforded flexibility in the course because they could work at their own pace, choose when and where to complete their out-of-class activities, and pause or rewind as many times as needed while watching video lectures. The STs also observed that English linguistic knowledge, which was included in video lectures, focused on the most critical points and was core and selected elements or essence of certain topics, which was seen as helpful to prepare and learn efficiently in the classroom. In the focus group interviews, one participant noted the efficiency of using FC:

*It's very time efficient…You can just do it whenever you want, and you can do it as quickly as you want.* (Participant_11_)

##### Repeatable usage of video lectures

The STs realized that it was easy for them to look back if they did not understand, and beneficial to repeatedly watch video lectures, especially when preparing for the exam in the course. Compared to traditional lectures in classroom, where the STs could only refer to their notes, the STs could re-watch the video lectures whenever they needed, which was also echoed in the interviews:

*I think it also makes me a bit calmer in this (exam) period that we can go back and watch them.* (Participant_14_)

##### Deep and collaborative learning in in-class activities

The STs noticed that by viewing video lectures during the out-of-class time, they might find out what they struggled with in advance so that during in-class time with FC, they could spend more time trying to understand those challenging parts. Moreover, the STs also found group and pair activities motivating, as one participant pointed out in the interviews:

*Most of us are prepared, and most of us have some ideas of what we didn't understand, then, we discuss them in smaller groups so we are more prepared with our questions with what we need more help with.* (Participant_4_)

##### Engaged teacher educators in in-class activities

The STs found it easier to ask the TE questions in FC because the TE visited and supported each group during the in-class time. They also got more time to ask questions and sensed that the in-class time was for getting help. Furthermore, they reported during the interviews that the TE had more time to answer their questions and clarify issues.

*We get some much more time to ask questions. I think I've asked much more questions in this semester than I have done in the last semester.* (Participant_15_)

##### Effective solution in Covid-19 pandemic

The STs regarded FC as an effective way of teaching during Covid-19. With less time in the classroom, they still learned a lot because they could watch video lectures in the out-of-class time and learn efficiently in group discussions when in the classroom. They stated in the interviews that:

*It’s also kind of helpful in the world situation where Corona is a thing.* (Participant_12_)

#### Challenges of FC perceived by student teachers

Among 34 survey participants, 67.65% of STs found that being unable to ask questions while viewing FC video lectures was challenging. 64.71% of STs thought that TEs could not know about their students’ preparation, and 55.88% of STs reported that their workload increased. Through open-ended questions in the survey, the STs stated that they might become “confused about new materials at home”, while working on out-of-class activities, such as viewing video lectures. Furthermore, they could not “ask questions right away”, and what they could do was to “either send an email (to the TE) or wait until class”. Since in-class activities in FC “depended on students coming prepared”, STs argued that it was “difficult to control if students have done the required work before class” and thus challenging for TEs to “make sure everyone meets up prepared”. For those who “come unprepared”, the STs noted that they could not “contribute equally in class”. In addition to reporting “a lot of readings and assignments”, the STs underscored that they should also “watch video lectures” in FC. Consequently, some sensed that “the workload on the students increases”.

**Fig. 3:**
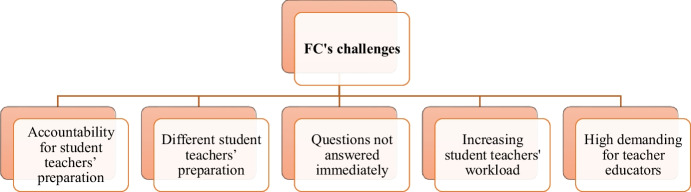
Challenges of flipped classroom

In the focus group interviews, the participating STs expressed their perceptions of the challenges of FC, which could be categorized into five themes, as presented in Fig. 3.

##### Accountability for student teachers’ preparation

The STs expressed that they had more responsibility in FC. The STs were required to come to class prepared. Otherwise, they argued that they could not actively participate in the in-class activities and could not learn as much. Some STs said they might not even show up to the classroom if they did not complete the out-of-class activities. One participant pointed out in the interviews that:

*With the flipped classroom, the preparation is half of the class, so when you don't prepare, you lose way more… It requires a lot of self-discipline.* (Participant_5_)

##### Different student teachers’ preparation

Since the STs were responsible for coming to class prepared, the level of preparation might vary. The STs noted in the interviews that they found it challenging when one or two or more peers in the same group could not contribute ideas in FC discussions. One participant noted that:

*What can happen is when you work in groups, the level might be on kind of different stages.* (Participant_2_)

##### Questions not answered immediately

While working on out-of-class activities alone and coming across some questions, the STs found it challenging because their questions could not be answered immediately. Instead, they stressed in the interviews that they had to wait until they returned to the classroom. Sometimes, they might have already forgotten their questions by then, as one participant stated:

*If there is something that you don't understand and you try to go back and back and back to look at it, then you have to wait maybe a long time to ask in class.* (Participant_9_)

##### Increasing student teachers’ workload

The STs also addressed that they had to spend much time working on out-of-class activities with FC. As to coming to the classroom prepared, the STs reported in the interviews that they spent time watching video lectures and reading materials in advance, as one participant emphasized:

*I spend one and a half hour on 20 minutes of video.* (Participant_13_)

##### High demanding for teacher educators

The STs noticed that FC demanded more of the TE compared to the *chalk and talk* way of teaching. The STs argued during the interviews that the TE needed to prepare video lectures and in-class activities and be ready for harder questions from STs during classroom time, which was echoed in the interviews:

*(The TE) really knows his subject and he's also a good facilitator, but both of those are highly needed for flipped classroom to be an efficient learning method.* (Participant_10_)

#### Immediate feedback on FC from student teachers

**Fig. 4:**
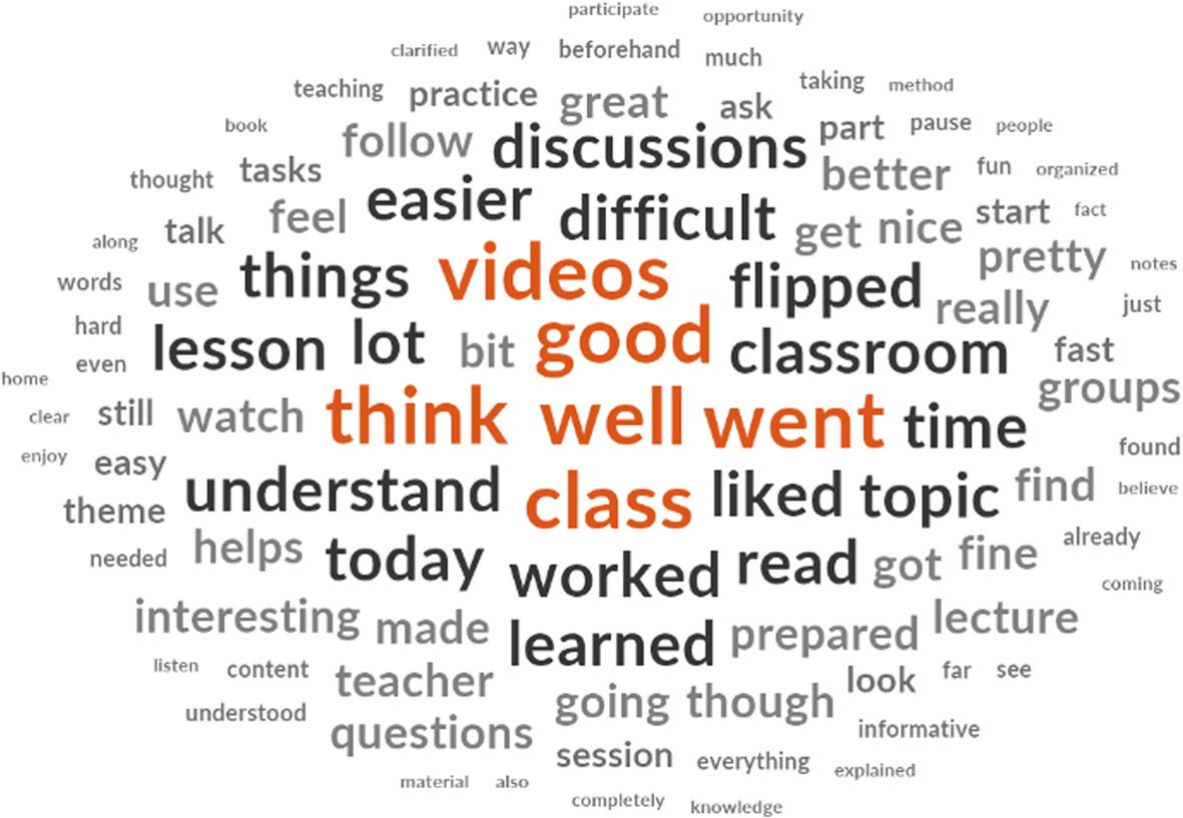
Immediate feedback on FC from student teachers

Figure 4 is a word cloud that visualizes the words that appeared most often across the exit tickets and provides an overview of STs’ immediate feedback on FC. As can be observed, “good” and “well” appeared more frequently than “difficult”. Meanwhile, “easier” and “liked” could also be noticed.

### Future flippers

After having experienced learning in FC, 70.59% of the 34 participating STs agreed or strongly agreed that they would like to take another course designed as FC. Furthermore, 79.41% of participants agreed or strongly agreed that with the learning experience of FC, they could do better in another FC course. However, compared with the willingness and fondness of taking another FC course by themselves, less than half of the participating STs (41.18%) agreed or strongly agreed that they would like to adopt FC in their teaching.

On the one hand, the STs stated that they would like to “apply different teaching approaches as students learn differently”. They believed that “with students ranging from almost fluent to struggling”, FC might “be easier for each student to adapt the materials to their own needs” and “open for more activities done in class”. On the other hand, as future teachers teaching in elementary or lower-secondary schools, the STs were concerned with FC’s utilization with “younger students”, because “the pupils are too young”, “need their teacher to be there physically”, and “might not be motivated enough by a video”.

In the focus group interviews, the STs communicated their perspectives on future flippers. From the interviews, all participating STs seemed to desire to take more FC courses. The STs also discussed the possibility of implementing FC in their teaching. However, some STs also mentioned that they might not implement FC in their teaching practice mainly due to the young age of their pupils (in Norway, first graders are at the age of 6).

*With all the benefits, I'd definitely like to have more flipped classroom courses.* (Participant_18_)

*Because they're too small, they're too young and they always expect that the teacher is going to elaborate and explain, so they won't be prepared, at least not at lower levels. But maybe for high school level, it could be beneficial.* (Participant_11_)

### Student teachers’ suggestions for flipped classroom

Based on the participating STs’ answers to the last question of the survey and their thoughts expressed through the focus group interviews, recommendations regarding three aspects for implementing FC were generated.

#### Suggestions on out-of-class activities

In line with their learning experience with FC, the participating STs suggested that video lectures should not be too long as out-of-class activities for students, and several short videos are better than a long one. The participants also emphasized creating variety in the out-of-class activities. However, various out-of-class activities should share a commonality: TEs need to be engaged to motivate their STs. In addition, the STs also suggested making space for pauses for students in the video lectures, and signal when they would like to have their students reflect. Even though their TE posted video lectures one week before physical classes, the participating STs would suggest viewing videos one or two days in advance to get clearer pictures in mind. Participants stated in the interviews that:

*It's more motivating to sit down and watch one video on 15 minutes now and I can watch the other one later.* (Participant_16_)

*There are many different types of presentations you can use, and to have variation is always good.* (Participant_14_)

#### Suggestions on in-class activities

With FC, lecturing time is moved out of the classroom, yet it does not mean that TEs cannot hold mini-lectures in the classroom. On the contrary, the participating STs suggested that TEs follow up on what STs did with video lectures, clear up potential misunderstandings after viewing video lectures, and repeat important information that needed attention during discussions or other activities. As one of the advantages brought by FC, the in-class activities could promote deeper and collaborative learning. Thus, TEs could prepare for more detailed or sophisticated questions from the STs and plan various student activities to promote their learning, such as group discussions. Furthermore, digital learning tools were suitable for out-of-class activities and fitting for in-class activities. Participants expressed that:

*Repeat some of the important things like in the class discussions or before the class discussions that you’ll be aware of what you have to focus on.* (Participant_8_)

*Like Padlets, not just during the digital lessons, but also in class, so the groups can write together, and it will come up on the smart board.* (Participant_11_)

#### Suggestions on courses suitable to be flipped

Since the participants were studying English language teaching, they emphasized several courses in their program that might be suitable for flipping, such as didactics, grammar, and phonetics. Meanwhile, the participating STs underlined some characteristics of a course suitable to be flipped. On the one hand, a course that contains complicated concepts or theories might be suitable for adopting FC. With the help of FC materials, such as video lectures, STs can review videos several times to better understand or assimilate complex information. STs can also take their questions and confusion to class to discuss with their peers or TEs. On the other hand, FC is suitable for a course that emphasizes incorporating activities, such as discussion or hands-on actions. With moving lectures out of class, STs can use longer in-class time for discussing or practicing, which also echoes the benefits perceived by participants arguing that FC can improve deep and collaborative learning. With courses that have the potential to be flipped, the STs underlined in the interviews a balance between flipped and non-flipped courses because of the increasing workload for them with the FC approach. Participants confirmed in the interviews that:

*It's nice to have the videos and see if there's something you don't understand, you can always go back and ease to check.* (Participant_6_)

*I like it, but I would not want to have this approach in every subject the same semester.* (Participant_3_)

## Discussion and conclusion

In this study, STs’ perceptions of Flipped Classroom were explored by analyzing data from the survey, focus group interviews, and exit tickets. The participants perceived both benefits and drawbacks of FC. Most participating STs agreed that FC was an effective teaching approach because FC used class time more efficiently. Therefore, FC could improve STs’ learning performance compared to the *chalk and talk* way of teaching. These findings support previous studies (e.g., González-Gómez et al., 2016; Jeong et al., 2018; Ng, 2018) that STs generally have positive perceptions of FC. Furthermore, this study categorized FC’s advantages perceived by STs into five aspects, as shown in Fig. 2. As a study conducted after the outbreak of Covid-19, the results of this study diverge to some degree from previous studies (e.g., Akçayır & Akçayır, 2018) regarding FC’s advantages. Yet, the study is also innovative because FC is suggested to be an effective solution during Covid-19. At present, the world is moving into a new post-pandemic phase. Moreover, hardly anyone knows whether something resembling a similar scenario could enforce remote teaching in schools and higher education. Based on the STs’ perceptions, FC is suggested as a pedagogical approach suitable in a pandemic or other critical situations, where remote teaching can be an alternative to physical teaching, such as during conflicts and natural disasters.

Besides FC’s advantages, over half of the participants found FC challenging for STs and TEs. In the study of Fraga and Harmon (2014), the STs mentioned time management and confusion as FC’s challenges. This study discovered the other two challenges for STs, i.e., they could not ask questions while viewing video lectures and experienced an increased workload. Apart from these challenges for STs, the participants thought that FC was also challenging for TEs since they were unable to know how the STs engaged in out-of-class activities. This study categorized FC’s challenges into five aspects, as Fig. 3 illustrates. On the one hand, these findings support the previous studies (e.g., Conner et al., 2014), but on the other hand, these findings also refer to FC’ challenges for TEs from the view of STs (e.g., Akçayır & Akçayır, 2018).

The STs’ opinions on being future flippers may predict what will happen in the future, in teacher and higher education and primary and secondary education. More than 70% of the participating STs would like to take another course designed as FC, and this result supports the previous studies (e.g., Dove & Dove, 2017; Jeong et al., 2016, 2018). Moreover, after getting familiar with FC, nearly 80% of the participants believed they could do better in another course with the FC approach. Therefore, educators in higher education, especially TEs in teacher education, may consider providing more courses with the FC approach.

However, as future teachers teaching in elementary or lower-secondary schools, only 40% of the participating STs wanted to adopt FC in their teaching career. This result is lower than Graziano’s study (2017), where most participating STs wanted to flip their classrooms in the future. The STs in this study are reluctant to implement FC in their classrooms mainly because of the age of their future pupils. In contrast, Graziano’s study (2017) participants were worried about the limited time. Nevertheless, there are STs in this study who would like to use FC or incorporate the approach in specific topics. These findings reveal that there may not be many future flippers among the participating STs, but FC may appear in some teachers’ classrooms in the future. Meanwhile, FC may be less desirable to implement in primary schools. Though none of the participants in this study and Graziano’s study (2017) mentioned a lack of PDC as a reason to refuse to implement FC in their teaching practice, a higher level of digital competence might help STs overcome those difficulties (Røkenes & Krumsvik, 2016; Erstad et al., 2021). If STs master the digital technologies required to produce a video lecture, with the possibility of reusing video lectures, STs can save time and energy in a long run. Furthermore, STs who have developed PDC in teacher education, such as through observing TEs’ modeling of FC in their coursework, are more likely to use technologies in a pedagogical and didactical manner to design and create out-of-class or in-class activities suitable for their future pupils’ age (Røkenes & Krumsvik, 2016; Guðmundsdóttir and Hatlevik, 2018).

As for suggestions for FC, the participating STs in this study proposed practical implications for TEs, some of which echo the previous studies (e.g., Conner et al., 2014). The STs suggested a couple of short video lectures instead of long ones and recommended pauses for students during video lectures. This idea shares the commonality with the STs’ suggestion of offering “a set of partially completed notes” (p. 73) in Conner et al.’s study in 2014. In addition to the length of video lectures, the STs emphasized the variety in the out-of-class activities and assessment forms. Besides PowerPoint presentations, Prezi presentations or podcasts were recommended. TEs needed to be engaged to motivate their STs, model pedagogical use of FC, and set aside time for student-centered in-class learning activities (Røkenes & Krumsvik, 2016; Røkenes et al., 2020). The STs recommended out-of-class activities that can promote critical reflection to support students as active thinkers and producers rather than as passive consumers of knowledge. Even though lecturing time is moved out of the classroom with the FC approach, the STs mentioned the necessity of including a mini-lecture or a recap during the in-class time. Moreover, the STs also advised TEs to be prepared for more detailed or sophisticated questions from STs and plan various in-class activities. For TEs who teach a course containing complex concepts or theories, it may be appropriate to consider implementing FC. Since the participants were studying English language teaching, they emphasized several courses in the subject discipline of English that might be suitable to be flipped, such as didactics, grammar, and phonetics. In addition, it is also advisable to consider the balance between the number of courses with and without FC.

The FC approach requires a different proficient level of digital competence of students and teachers, such as PDC (Krumsvik, 2011; Lund et al., 2014). FC demands students’ basic digital skills to use learning management systems, laptops, and interactive learning tools and requires a higher level of digital competence of teachers (Røkenes & Krumsvik, 2016). With the FC approach, teachers’ “didactic ICT-competence” and “learning strategies” are needed to reflectively and pedagogically use ICT and seamlessly integrate ICT in preparing video lectures and understand ICT’s impact on learning environment and assessment forms (Røkenes & Krumsvik, 2016). On the one hand, for those STs who would like to become future flippers, it is valuable to develop their digital competence and utilize the FC approach in their teaching. On the other hand, STs reluctant to implement FC need to continuously develop their PDC for meeting future scenarios and emerging technologies, such as dealing with the artificial intelligence software ChatGTP and plagiarism. The study of Jimoyiannis and Koukis (2023) also confirmed that “the role of digital technologies in education will be more important” (p. 13) after Covid-19. Therefore, it is valuable to highlight digital competence in education. The FC approach is potentially helpful as an approach requiring the digital competence of both teachers and students.

## Limitation and future research

This study examined students’ perceptions of FC through evidence from STs. The participants in this study were all from an EFL teacher education program at a university in Norway. The conclusions might be more reliable and generalizable if the participants were more diverse, such as from different subject disciplines and teacher education institutions. In addition, this study investigated a course with the FC approach over one academic semester. Future studies should address various subject disciplines, including participants from several teacher education programs, and examine the long-term effects of implementing FC. Furthermore, since some STs are concerned with implementing FC in primary schools, conducting further research of FC in primary education is advisable.

## Figures and Tables

**Table 2:**
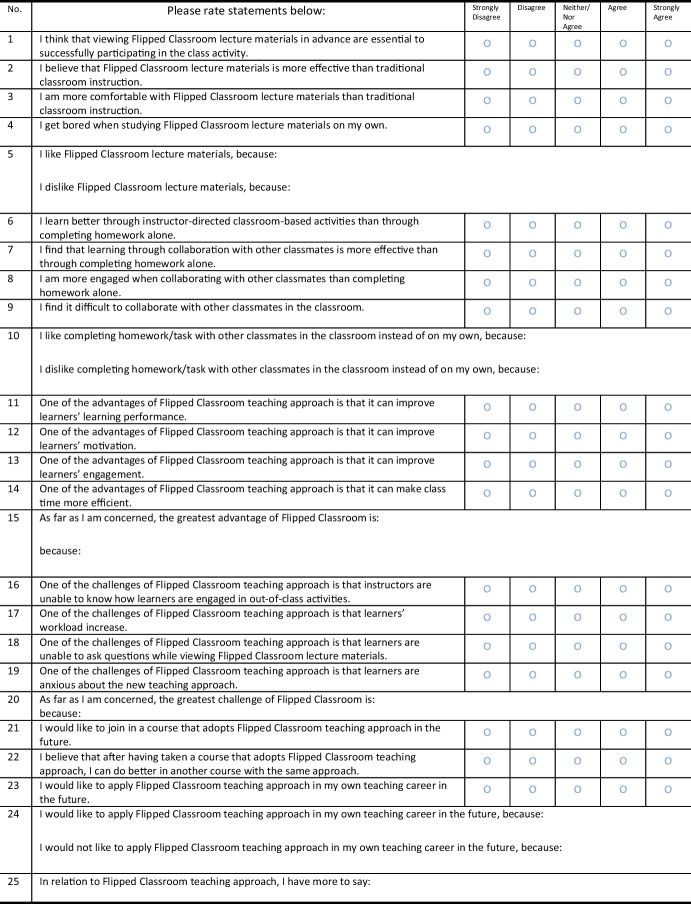
Survey on perceptions of flipped classroom teaching approach

## Data Availability

The data supporting this study’s findings are available on request from the corresponding author H. H. The data are not publicly available because they contain information that could compromise research participant privacy.

## Appendix 1

2

## Appendix 2

**Table 3:** Guideline for focus group interview

Warm-up
• When and how did you first encounter Flipped Classroom? ○ Please elaborate on what the activity was and what you thought about flipped classroom the first time you encountered it
Perceptions
• How do you think the online video lectures impacted your learning? • How do you think group discussions and teamwork impacted your learning compared to listening to lectures and doing individual work? • What do you believe are some of the benefits of using Flipped Classroom in this course? • What do you believe are some of the drawbacks of using Flipped Classroom in this course? ○ What solutions do you suggest solving the problems experienced?
Future teaching
• How do you like having more courses with Flipped Classroom teaching? ○ In English language teaching, do you think that there are topics that are work better than others using Flipped Classroom (e.g., grammar, literature, vocabulary, reading, writing etc.)? ○ What advice would you give another teacher who was thinking of making a similar change to one of his or her courses? • How do you like using Flipped Classroom in your own teaching?

3

## Footnotes

1 In Norway, grades 1–7 are elementary schools and grades 8–10 are lower-secondary schools.

2 https://www.socrative.com/
